# The Combination Options and Predictive Biomarkers of PD-1/PD-L1 Inhibitors in Esophageal Cancer

**DOI:** 10.3389/fonc.2020.00300

**Published:** 2020-03-05

**Authors:** Hui Yang, Kunlun Wang, Tao Wang, Mengxi Li, Bingxu Li, Shenglei Li, Ling Yuan

**Affiliations:** Department of Radiation Oncology, The Affiliated Cancer Hospital of Zhengzhou University, Zhengzhou, China

**Keywords:** esophageal cancer, programmed death-1, programmed death-ligand 1, combination therapy, predictive biomarkers

## Abstract

Esophageal cancer (EC) is one of the most common cancers with poor survival in the world. Nowadays, a generous number of clinical trials are underway on the use of immunotherapy in EC patients, especially the programmed death-1/programmed death-ligand 1 (PD-1/PD-L1) inhibitors. However, only a few patients could benefit from single-agent therapy. Others need combination therapies to enhance the response rate and survival. In this review, we focus on PD-1/PD-L1 inhibitors and its combination options in EC patients. We also summarized the potential predictive biomarkers for PD-1/PD-L1 inhibitors treatment.

## Introduction

Esophageal cancer (EC) is the seven most common cancer and ranks second in the cause of cancer-related death worldwide (1). It comprises esophageal squamous cell carcinoma (ESCC) and esophageal adenocarcinoma (EAC), with different pathogenesis and distribution. ESCC accounts for ~90% of EC in Asia and has a close relationship with smoking, having hot food or water and alcohol consumption (2). Whereas, EAC is the dominant type in western countries and is usually caused by chronic gastroesophageal reflux disease (GORD), obesity and Barrett's esophagus (3). Esophagogastric junction (EGJ) adenocarcinoma is often grouped with EAC because of their similar etiology.

Traditional therapies for EC patients include surgery, chemotherapy, radiotherapy (RT), and targeted therapy. However, most patients relapse quickly after the initial therapy. Meanwhile, EC patients always have a lack of oncogenic driver mutations (4), and the addition of the targeted drugs can only prolong survival for a few months (5). Hence, novel drugs with definitive efficacy to improve overall survival (OS) are expected.

In recent clinical trials of EC, interest is very high in immunotherapies, which involved immune checkpoint inhibitors (ICIs), adoptive T-cell therapy, cancer vaccines, and oncolytic viruses. Immunotherapies act on different steps of the anti-tumor immunity to enhance the host's immunity and strengthen anti-tumor responses. A series of events occurred in steps to eliminate cancer cells and were identified as the cancer-immunity cycle (6). First, tumor-specific antigens are specifically recognized by dendritic cells (DCs) or antigen-presenting cells (APCs) (step 1). After that, antigens are presented to T cells (step 2) and participate in the priming and activation of effector T cells (step 3). Next, activated effector T cells traffic to (step 4) and infiltrate to cancer cells (step 5). Then, effector T cells recognize (step 6) and finally kill the cancer cells (step 7). The death of cancer cells further results in more release of tumor-specific antigens (step 1 again) and strengthens the cancer-immunity cycle. However, cancer cells could evade immune surveillance through various mechanisms, including faulty recognition of neoantigens, inhibition of T-cell infiltration and suppression of effector T cells (7).

ICIs, especially programmed death-1/programmed death-ligand 1 (PD-1/PD-L1) inhibitors, have demonstrated clinical benefit in multiple cancers and generated tremendous interest. Immune checkpoints are immunosuppressive proteins and help to maintain immunologic homeostasis in hosts (8). Interaction of ICI with checkpoint breaks immunosuppression and enhances anti-tumor immunity (9). PD-1/PD-L1 inhibitors are the most well-known immune checkpoints. The PD-L1 on the surface of tumor cells binds to PD-L1 on cancer cells, and reduce the function of cytotoxic T lymphocyte (CTL), inhibit the anti-tumor function of T cells, and lead to immune escape in the effector phase of the cancer-immunity cycle (10).

We summarize the clinical studies of PD-1/PD-L1 inhibitors and the combination options for EC in this review.

## PD-1/PD-L1 Inhibitors in EC

PD-L1 overexpression has been reported in around 40% of EC patients and is related to worse OS (11). Various methods could be used to detect PD-L1 expression in different cancers, including immunohistochemistry (IHC) staining, enzyme-linked immunosorbent assay (ELISA), immunofluorescence (IF), flow cytometry (FC) and so on. IHC is the most common and convenient one. Now, four diagnostic kits are commercially available: the 22C3 and 28-8 clones of Dako Autostainer Link 48 platform^1^^,^ ^2^; the SP263 and SP142 clones of Ventana BenchMark ULTRA platform^3^^,^ ^4^. The PD-L1 antibody used in IHC staining is the Dako 22C3 pharmDx (12). Comparison studies of different PD-L1 antibodies are urgently needed.

Furthermore, combined positive score (CPS) is used to define the PD-L1 status instead of the tumor proportion score (TPS) for EC patients. CPS is defined as the ratio of the combining number of PD-L1 positive tumor cells and immune cells (lymphocytes, macrophages) by IHC staining to the total number of tumor cells. The maximum score is 100, and a higher score showing greater likelihood of response to PD-1/PD-L1 inhibitors (12).

The most studied PD-1/PD-L1 inhibitors were Nivolumab (OPDIVO, Bristol-Myers Squibb Co.), Pembrolizumab (KEYTRUDA, Merck Sharp & Dohme Co., Inc.), Avelumab (BAVENCIO, EMD Serono Inc.), Atezolizumab (TECENTRIQ, Genentech Inc.), and Durvalumab (IMFINZI, AstraZeneca Inc.). Plenty of new PD-1/PD-L1 inhibitors are being investigated now, including PD-1 inhibitors: Camrelizumab (SHR-1210, Jiangsu HengRui Medicine Co., Ltd.), Sintilimab [IBI308, Innovent Biologics (Suzhou) Co. Ltd.], Spartalizumab (PDR001, Novartis Pharmaceuticals), Tislelizumab (BGB-A317, BeiGene), Toripalimab (triprizumab, teripalimab, JS001, Shanghai Junshi Biosciences Co., Ltd), HLX-10 (Shanghai Henlius Biotech, Inc.) and PD-L1 inhibitor: SHR-1316 (Jiangsu HengRui Medicine Co., Ltd.) and CS1001 (CStone Pharmaceuticals Co., Ltd.).

## The Ongoing Clinical Trials in EC

ICIs have shown considerable objective response rates (ORR), durable toxicities and even prolong the OS in several cancers, including advanced EC. However, the effective rates of single-agent were 31% in melanoma and only 17% in lung cancer, respectively (13). And the response rate of ICI alone in EC patients varied from 9.9 to 33.3% in the reported studies (14). The combination of PD-1/PD-L1 inhibitors with other therapies, such as chemoradiotherapy (CRT), other ICIs, cancer vaccines, and target drugs, was supposed to make the tumor more immunogenic, produce a synergistic effect, and gather stronger clinical benefit.

We searched the ClinicalTrials.gov database with the search terms “immunotherapy,” “esophageal cancer,” “PD-1,” “PD-L1,” and their variants. Then we screened the search results and recorded the clinical trial numbers. With the numbers, we obtained relevant articles from PubMed, Embase, American Society of Clinical Oncology (ASCO), ASCO Gastrointestinal Cancers Symposium, European Society for Medical Oncology (ESMO) and World Organization for Specialized Studies on Diseases of the Esophagus (OESO) databases. All the trials of PD-1/PD-L1 inhibitors in EC patients with different stages were included (Tables 1, 2), and we focused on trials with published results in this review. The detailed information of published trials were shown in Table S1.

**Table 1:** The combination with PD-1 inhibitors in EC.

**Drug**	**Neoadjuvant**	**Adjuvant**	**First-line**	**Second-line or subsequent**
Nivolumab	NCT03044613 (CRT + Relatlimab) NCT02946671 (Mogamulizumab) NCT03278626 (CRT) NCT03544736 (CRT) NCT03604991 (CRT ± Ipilimumab) NCT03914443 (Chemo)	NCT02743494 (After CRT + surgery)	NCT3143153 (Ipilimumab/chemo) NCT03437200 (CRT ± Ipilimumab) NCT03544736 (CRT/Chemo)	NCT02476123 (Mogamulizumab) NCT03416244 (Ipilimumab) NCT03241173 (INCAGN01949 ± Ipilimumab) NCT03544736 (Palliative RT for primary tumor)
Pembrolizumab	NCT02844075 (CRT) NCT02998268 (CRT/chemo) NCT03064490 (CRT) NCT03322267 (CRT) NCT03592407 (Epacadostat) NCT03792347 (CRT)	NCT02844075 (After CRT + surgery) NCT03322267 (After CRT + surgery)	NCT03189719 (Chemo) NCT03881111 (Chemo)	NCT02013154 (DKN-01) NCT02642809 (Brachytherapy: 16Gy/2F) NCT02830594 (Palliative RT) NCT03277352 (INCAGN01876 + Epacadostat) NCT03993353 (Tadalafil)
Camrelizumab	NCT03200691 (RT) NCT03917966 (Chemo)	NCT03817658 (After CRT) NCT03985046 (After CRT)	NCT03187314 (RT) NCT03222440 (RT) NCT03603756 (Apatinib ± chemo) NCT03671265 (CRT) NCT03691090 (Chemo)	NCT03736863 (Apatinib) NCT03766178 (Nimotuzumab)
Sintilimab	NCT03940001 (CRT) NCT03946969 (Chemo)		NCT03748134 (Chemo)	
Spartalizumab				NCT01351103 (LGK974) NCT02460224 (LAG525) NCT03365791 (LAG525) NCT03785496 (MCS110) NCT04000529 (TNO155)
Tislelizumab			NCT03469557 (Chemo) NCT03783442 (Chemo) NCT03957590 (CRT)	
Toripalimab	NCT03985670 (Chemo) NCT04006041 (CRT)		NCT03829969 (Chemo) NCT04005170 (CRT) NCT04084158 (CRT)	
HLX-10			NCT03958890 (Chemo)	

**Table 2:** The combination with PD-L1 inhibitors in EC.

**Drug**	**Neoadjuvant**	**Adjuvant**	**First-line**	**Second-line or subsequent**
Avelumab	NCT03490292 (CRT)		NCT03490292 (CRT) NCT03800953 (CRT)	
Atezolizumab	NCT03087864 (CRT) NCT03784326 (CRT)	UMIN000034373 (After CRT)	NCT03087864 (CRT)	NCT03170960 (Cabozantinib) NCT03818997 (DKN-01 ± chemo) NCT03829501 (KY1044)
Durvalumab	NCT02735239 (CRT/Chemo) NCT02962063 (CRT)	NCT02639065 (After CRT + surgery) NCT02520453 (After CRT + surgery) NCT03377400 (+Tremelimumab, after CRT) NCT04054518 (After CRT)	NCT02658214 (Chemo + Tremelimumab) NCT03377400 (CRT + Tremelimumab) NCT03777813 (CRT)	NCT02735239(Chemo ± Tremelimumab) NCT03212469 (Tremelimumab + SBRT) NCT03292250 (Tremelimumab) NCT03982173 (Tremelimumab)
SHR-1316			NCT03732508 (Chemo)	NCT03766178 (Nimotuzumab)

### As Neoadjuvant Treatment

The CROSS study showed that neoadjuvant concurrent CRT induced 23% pathologic complete response (pCR) and prolong median overall survival (mOS) (49 vs. 24 months; hazard ratio (HR) = 0.657, 95% confidence interval (CI): 0.495–0.871, *p* = 0.003) without extra toxicities compared with surgery alone (15). Now, neoadjuvant CRT followed by surgery is the standard treatment for resectable locally advanced EC patients. However, up to 50% of patients relapsed in one year after surgery and the 5-year survival is only 43% (16). Alternative treatments are needed to further improve the survival outcomes with durable toxicity.

According to preclinical studies of EC, RT could induce immunogenic cell death (ICD), consequently release neoantigens, alter tumor microenvironment (TME), and finally activate the immune response (17). Besides, the expression of PD-L1 and CD8+ CTLs in TME could be upregulated by prior CRT. In turn, ICIs also provide synergistic effect to RT through targeting and modulating various T cells population. A growing number of clinical trials are preforming now to evaluate the safety and efficacy of combining CRT with PD-1/PD-L1 inhibitors before surgery (Table 3).

**Table 3:** The published data of neoadjuvant or adjuvant use of PD-1/PD-L1 inhibitors.

**Drug**	**Study**	**Role**	**Patients**	**Disease**	**Chemotherapy**	**RT**	**pCR**	**Pneumonitis**
Nivoluzumab	NCT03044613	Neoadjuvant	10	EAC	Paclitaxel + carboplatin, q1w	RT	40.0%	0
Pembrolizumab	NCT02844075	Neoadjuvant and adjuvant	26	ESCC	Paclitaxel + carboplatin, q1w	44.1 Gy/21 fr	46.1%	–
Avelumab	NCT03490292	Neoadjuvant	6	EAC	Paclitaxel + carboplatin, q1w	41.4 Gy/23 fr	43.0%	–
Atezolizumab	NCT03087864	Neoadjuvant	23	EAC	Paclitaxel + carboplatin, q1w	41.4 Gy/23 fr	39%	–
Atezolizumab	UMIN000034373	Adjuvant	56	ESCC	Cisplatin + fluoracil, q4w, two cycles	60 Gy/30 fr	–	–
Durvalumab	NCT02639065	Adjuvant	24	EAC or GEJ	Carboplatin + paclitaxel/cisplatin + fluoropyrimidine, surgery	RT	–	1 (grade 3)

Nivolumab is a humanized IgG4 monoclonal PD-1 antibody. Its neoadjuvant role was assessed in the trial NCT03044613. This study recruited 16 EC patients to receive two cycles of induction nivolumab before CRT and three additional cycles of nivolumab concurrently with CRT (18). The esophagectomy was performed 6–10 weeks after the last nivolumab. To date, ten EAC patients have had surgery, and the pCR was 40% (4/10). Combination therapy has acceptable toxicity and did not delay the surgery. Toxicities of note include steroid-responsive grade 3 dermatitis (1/16) and grade 3 hepatitis (1/16).

Pembrolizumab is another humanized IgG4 monoclonal PD-1 antibody. The trial NCT02844075 enrolled 28 ESCC patients to receive neoadjuvant CRT plus pembrolizumab (19). Twenty-six patients performed esophagectomy in 5 weeks after the neoadjuvant treatment completed. Two patients died after surgery because of acute lung injury. After surgery, patients were treated with pembrolizumab for up to 24 months or until progression, death or unacceptable toxicity. The pCR in the primary tumor was 46.1% (12/26, 95% CI: 28.8–64.6) and the 1-year survival rate was 80.8% (mOS has not been reached). The common treatment-related adverse events (trAEs) were neutropenia (50.0%) and increased liver enzyme (30.8%). There was a tendency toward better disease-free survival (DFS) in patients reached pCR in the primary tumor (HR = 0.33, *p* = 0.1).

As for PD-L1 inhibitors, the trial NCT03490292 tested the safety and efficacy of avelumab with CRT in esophageal or EGJ adenocarcinoma patients (20). Avelumab was given after the last dose of chemotherapy on day 29. Surgery was performed 8 weeks after CRT completion and patients received eight cycles of avelumab after resection. One EAC, three Siewert I EGJ and two Siewert II EGJ cancer patients were enrolled. Five patients underwent R0 resection and one patient had R1 resection because tumor had invaded to the adventitial surface. In this trial, pCR was 43% and no dose-limited toxicity (DLT) or grade ≥ 3 immune-related adverse events (irAEs).

In another phase II study, PERFRCT trial (NCT03087864), resectable EAC patients were enrolled to receive five cycles of concurrent CRT and atezolizumab before surgery (21). So far, 39 patients were recruited and 23 patients have had R0 resection. The pCR was 39% (9/23). Grade 3–4 adverse events (AEs) occurred in 48.4% (15/31) patients and were all manageable. There was no report of surgery delay.

### As Adjuvant Treatment

The value of postoperative chemotherapy in resectable esophageal and EGJ cancers remains uncertain in the previous trials (22–24). After R0 resection, observation is advised for ESCC patients by National Comprehensive Cancer Network (NCCN) guidelines (25). However, NCCN guidelines recommend either chemotherapy or observation for EAC patients who received preoperative CRT and surgery.

For unresectable locally advanced ESCC, the definitive CRT and observation after that is the standard treatment. However, the complete response (CR) rate is only 11 to 25%, 1-year relapse-free survival (RFS) rate is 50.0% and mOS is only 9–10 months (26). The increase of radiation dose or addition of any adjuvant treatment could not improve the local control rate or provide a survival benefit (27, 28). Based on the result of PACIFIC trial (NCT02125467), durvalumab made an 11-month advantage in progression-free survival (PFS) over placebo (16.8 vs. 5.6 months; HR = 0.52, 95% CI: 0.42–0.65) and better OS (HR = 0.69, 95% CI: 0.55–0.86) as adjuvant treatment after definitive CRT in stage III non-small cell lung cancer (NSCLC) (29, 30).

The adjuvant role of PD-1/PD-L1 inhibitors in EC have been reported in the 2019 ASCO meeting. A phase II trial, NCT02639065 was designed for patients with resected locally advanced esophageal or EGJ adenocarcinoma who had a viable tumor in the surgical specimen after neoadjuvant CRT and R0 resection (31). Enrolled 24 patients received durvalumab for up to 12 months after CRT and surgery. The median number of adjuvant durvalumab cycles was 12.5 (range: 2–13). Three patients developed grade 3 irAEs, one each with pneumonitis, hepatitis, and colitis. At data cutoff, seven (29%) patients relapsed, six (25%) patients had a distant relapse (lung, brain, bone, cervical lymph nodes) and one (0.4%) patient had a locoregional relapse. The 1-year RFS rate, 1-year survival rate, and 2-year survival rate were 79.2, 95.5, and 59.2%, respectively. Median survival time after relapse was 11.1 months (95% CI: 0.1–11.3 months).

In the TENERGY trial (UMIN000034373), unresectable locally advanced ESCC patients without distant metastasis were enrolled and treated with atezolizumab for up to 12 months within 4 weeks after two cycles definitive CRT (32). So far, 50 patients have been enrolled to evaluate the adjuvant role of atezolizumab.

Based on the published data, the addition of PD-1/PD-L1inhibitors as neoadjuvant or adjuvant treatment demonstrated promising efficacy with acceptable toxicity. The trials are ongoing with camrelizumab, sintilimab, and toripalimab as showed in Table 1. Further studies are awaited to identify the most beneficial patients according to PD-L1 status and so on.

### As First-Line Treatment

First-line platinum-based doublet chemotherapy provides a limited survival benefit in advanced ESCC patients. To gain better survival, an effective combination with other therapy is urgently required.

#### Combination With RT

Several studies evaluated the efficacy of PD-1/PD-L1 inhibitors plus RT as first-line treatment in advanced EC patients. As mentioned before, RT could enhance the anti-tumor immunity and induce a synergistic effect with PD-1/PD-L1 inhibitors.

The combination of RT with camrelizumab has been tested in a phase II, single-arm study for patients with locally advanced EC intolerant to or refused CRT (NCT03187314) (33). Sixteen patients were treated with camrelizumab (5 cycles) and RT (60 Gy/30 fr) as first-line treatment. One (7.1%) patient had CR and 13 (92.9%) patients had a partial response (PR). At the data cut off, two patients had metastasis, and median survival had not been reached.

Another phase Ib trial NCT03222440 evaluated camrelizumab with RT as first-line therapy in 20 ESCC patients and observed two (11.1%) patients had CR and 13 (72.2%) patients with PR (34). Patients were treated with RT (60 Gy/30 fr) and concurrent camrelizumab (from the start of RT, up to 16 cycles). Two (11.1%) patients had CR, 13 (72.2%) patients had PR, and three (16.7%) patients had stable disease (SD).

#### Combination With Chemotherapy

Chemotherapy could also facilitate the anti-tumor response. When combined with immunotherapy, chemotherapy could promote the presentation of tumor antigen, enhance the filtration of CTL and improve the efficacy of checkpoint inhibition (35, 36).

The phase II trial, NCT03469557 evaluated the tolerability of tislelizumab combined with chemotherapy (cisplatin and fluorouracil) as first-line treatment in Chinese patients with inoperable, locally advanced ESCC (37). A total of 15 patients were enrolled and grade ≥ 3 AEs occurred in eight patients, including one grade 5 hepatic dysfunction. Four patients discontinued treatment because of AEs, including grade 3 tracheal fistula, grade 3 lung infection, grade 2 pneumonitis, and grade 3 aspartate aminotransferase (AST) increasing. The efficacy data remains unmatured.

The phase III study KEYNOTE-590 (NCT03189719, MK-3475-590) of pembrolizumab plus chemotherapy (cisplatin and fluorouracil) as first-line therapy for advanced or metastatic EC is ongoing. Its China Extension study, NCT03881111 is also underway now.

#### Combination With ICIs

Since the different mechanisms of ICIs, the combination of PD-1/PD-L1 inhibitors with other ICIs may be significant. Cytotoxic T-lymphocyte-associated protein 4 (CTLA-4) on T cells interact with peripheral membrane B7 on APCs and impede the activation of T cells (38). So, CTLA-4 inhibitors (ipilimumab or tremelimumab) act on the antigen presentation phase to induce CTL and produce a synergistic effect with PD-1/PD-L1 inhibitors. CTLA-4 inhibitors are the most frequent combination option and yielded enhanced responses with a manageable safety profile in multiple cancers.

The phase I trial of NCT02658214 evaluated the safety of durvalumab and tremelimumab in combination with chemotherapy (cisplatin and fluorouracil) as first-line treatment for advanced ESCC patients (39). Six patients were recruited to receive four cycles of combination treatment. Four patients had recurrent disease and two were newly diagnosed with advanced ESCC. Three patients had grade ≥ 3 chemotherapy-related AEs (two grade 2 neutropenia and one grade 4 neutropenia). Two patients had irAEs and were grade 1 or 2 diarrhea, pruritus, rash, and increased AST. There were no trAEs-related discontinuation or death. Two of the six patients had a confirmed PR at data cutoff.

The trial CheckMate 648 (NCT03143153) was a randomized, phase III study to compare nivolumab plus ipilimumab or nivolumab combining with chemotherapy (cisplatin and fluorouracil) vs. chemotherapy alone as first-line treatment in unresectable, recurrent, or metastatic ESCC patients. Future results will provide more safety and efficacy data on the combination of PD-1/PD-L1 inhibitors and CTLA-4 inhibitors.

#### Combination With Target Drugs

NCT03603756 was a phase II study of camrelizumab combined with apatinib [a vascular endothelial growth factor receptor 2 (VEGFR2) inhibitor] and chemotherapy (liposomal paclitaxel and nedaplatin) as the first-line treatment for advanced ESCC (40). 30 patients received six to nine cycles of combination treatment, followed by maintenance therapy (camrelizumab, apatinib, or both). The ORR was 80.0% (24/30) and the disease control rate (DCR) was 96.7% (29/30). PFS and OS data had not matured. The most common grade 3–4 AEs were leucopenia (83.3%) and neutropenia (60.0%). The incidence of capillary hemangiomas was dramatically decreased because of the inhibition effect of apatinib to VEGFR2, which made their combination more reasonable.

### As Second-Line or Subsequent Treatment

For patients who failed in the treatment of standard platinum-based doublet chemotherapy, second-line, and subsequent treatment options are limited. The 5-year survival rate was ~20% in all stages and only 5% in advanced patients (41). The mOS is always shorter than one year in metastatic patients (42). The safety and efficacy of PD-1/PD-L1 inhibitors were assessed in tremendous clinical trials (Table 4).

**Table 4:** The published data of PD-1/PD-L1 inhibitors in advanced EC.

**Drug**	**Study**	**Design**	**Patients**	**Disease**	**ORR**	**DOR(m)**	**mPFS(m)**	**mOS(m)**
Nivolumab	ATTRACTION-1	II, single-arm, second-line	65	Japanese ESCC	17.2%	11.17	1.5	10.8
	ATTRACTION-3	III, randomized controlled, second-line	210	ESCC	19.0%	6.9	1.7	10.9
Pembrolizumab	KEYNOTE-028	II, single-arm, second-line or subsequent	23	EC	30%	15.0	1.8	7.0
			18:5	ESCC vs. EAC	28%vs. 40%	–	–	–
	KEYNOTE-180	II, single-arm, second-line or subsequent	121	EC	9.9%	Not reached	2.0	5.8
			63:58	ESCC vs. EAC	14.3 vs. 5.2%	Not reached	–	–
			58:63	PD-L1 (+) vs. (–)	13.8 vs. 6.3%	Not reached	–	–
	KEYNOTE-181	III, randomized controlled, second-line	–	EC	–	–	–	7.1
			–	ESCC	16.7%	9.3	–	8.2
			107	PD-L1 (+)	21.5%	9.3	2.6	9.3
			85:22	PD-L1 (+) ESCC vs. EAC	22.0 vs. 18.0%	9.3 vs. Not reached	3.2 vs. 2.1	10.3 vs. 6.3
			62	Chinese EC	16.1%	Not reached	–	8.4
Camrelizumab	NCT02742935	I, single-arm, second-line or subsequent	30	Chinese ESCC	33.3%	–	3.6	–
	NCT03099382	III, randomized controlled, second-line	228	Chinese ESCC	20.2%	–	–	8.3
Toripalimab	NCT02915432	I/II, single-arm, second-line or subsequent	59	Chinese ESCC	18.6%	11.2	–	–

#### PD-1/PD-L1 Inhibitors Alone

##### Nivolumab

ATTRACTION-1 (ONO-4538-07/JapicCTI-No.142422) study was a single-arm, phase II study enrolled 65 Japanese ESCC patients with unresectable or recurrent EC and were refractory to or intolerant to standard chemotherapy (fluoropyrimidine and platinum) (43). In this trial, patients were unselected with PD-L1 expression and received second-line nivolumab. ORR was 17.2% (95% CI: 9.9–28.2%) with three CR and eight PR. The reported median duration of response (mDOR) was 11.17 months (95% CI: 3.02-not reached). The median progression-free survival (mPFS) was 1.5 months (95% CI: 1.4–2.8 months) and mOS was 10.8 months (95% CI: 7.4–13.3 months). Thirty-nine patients (60%) had trAEs, and grade 3 or 4 AEs happened in 14 and 3% of patients, respectively. Diarrhea (14%) and decreased appetite (9%) were the most common ones. Treatment discontinuation occurred in seven (11%) patients due to lung infection, decreased appetite, interstitial lung disease, hepatic function abnormal, hyponatremia, dyspnoea, and eosinophilic pneumonia. There was no treatment-related death.

Furthermore, the randomized, phase III trial, ATTRACTION-3 (NCT02569242, ONO-4538-24/CA209-473) is performing to compare nivolumab with chemotherapy (docetaxel or paclitaxel) as second-line therapy in unresectable or recurrent ESCC patients with PD-L1-unselected (44). So far, 419 patients were randomized and 96% (401/419) were Asian patients. ORR was 19% (95% CI: 14–26.0%) and mDOR was 6.9 months (95% CI: 5.4–11.1 months) in the nivolumab group (210 patients). Compared with chemotherapy group, the nivolumab group showed better mOS (10.9 vs. 8.4 months; HR = 0.77, 95% CI: 0.62–0.96; *p* = 0.02) (45). The mPFS didn't display a statistically significant difference (1.7 vs. 3.4 months; HR = 1.08, 95% CI: 0.87–1.34). Fewer trAEs were reported in the nivolumab group (any grade, 66 vs. 95%; grade 3–4, 18 vs. 63%).

##### Pembrolizumab

KEYNOTE-028 (NCT02054806) was a single-arm, phase Ib trial that enrolled PD-L1 positive (CPS > 1) locally advanced or metastatic EC patients to receive pembrolizumab (12). Among the 23 enrolled patients, 18 (78%) patients were ESCC, and others were EAC. ORR was 30% (95% CI: 13–53%) in all patients, 28% in ESCC patients and 40% in EAC patients. And the mDOR was 15 months (95% CI: 6–26 months). Overall, mPFS was 1.8 months (95% CI: 1.7–2.9 months) and mOS was 7.0 months (95% CI: 4.3–17.7 months). Only nine patients (39%) experienced trAEs, and grade 3 trAEs occurred in four patients (17%). There was no grade 4 trAE, death or discontinuation in this trial. KEYNOTE-028 firstly demonstrated the manageable toxicity and durable anti-tumor activity of pembrolizumab in EC.

KEYNOTE-180 (NCT02559687) was a single-arm, phase II study which enrolled patients with locally advanced or metastatic ESCC and EAC (including Siewert type I EGJ adenocarcinoma) who refractory to at least two prior systemic treatments (46). One hundred twenty-one patients were enrolled with unselected PD-L1 expression. 63 (52.1%) patients were ESCC and 58 (47.9%) patients had PD-L1 positive (CPS ≥ 10). ORR was 9.9% (two CR and ten PR) and mDOR was not reached (1.9–14.4 months). The mPFS was 2.0 months (95% CI: 1.9–2.1 months) and mOS was 5.8 months (95% CI: 4.5–7.2 months) in all patients. ORR was higher in ESCC subgroup (14.3 vs. 5.2%), and better in PD-L1 positive subgroup (13.8 vs. 6.3%). In the 35 PD-L1 positive ESCC patients, ORR was 20.0% (95% CI: 8.0–37.0) and duration of response (DOR) varied from 4.2 to 25.1+ months, with 14.3% (5/35) patients being effective for over 6 months and 8.6% (3/35) patients having responses more than 12 months. Overall, 19 (15.7%) patients had grade 3–5 trAEs. Only seven patients discontinued due to AEs and one patient died of pneumonitis.

Since pembrolizumab was certified as an effective and safe third-line treatment in the trial KEYNOTE-028 and KEYNOTE-180, the trial KEYNOTE-181 (NCT02564263) evaluated its upfront use as second-line treatment. The trial enrolled 628 patients with locally advanced or metastatic EC who progressed on or after the standard chemotherapy (47). Patients were randomized to receive either pembrolizumab or chemotherapy: paclitaxel, docetaxel or irinotecan. In all the patients, the pembrolizumab group did not display better OS (7.1 vs. 7.1 months; HR = 0.89, 95% CI: 0.75–1.05, *p* = 0.0560). However, in the 222 (35.4%) patients with PD-L1 positive (CPS ≥ 10), ORR was higher (21.5 vs. 6.1%, *p* = 0.0006) and DOR was longer (9.3 vs. 7.7 months) in pembrolizumab as compared with chemotherapy. Hence, pembrolizumab could meaningful improve the OS as the second-line therapy compared with chemotherapy in PD-L1 positive (CPS ≥ 10) patients (mOS: 9.3 vs. 6.7 months; HR = 0.69, 95% CI: 0.52–0.93, *p* = 0.0074). For the 401 ESCC patients, ORR (16.7 vs. 7.4%, *p* = 0.0022) was better in the pembrolizumab group and the mOS was 8.2 months (95% CI: 6.7–10.0 months) in the pembrolizumab group and 7.1 months (95% CI: 6.1–8.2) in chemotherapy group (HR: 0.78, 95% CI: 0.63–0.96, *p* = 0.0095). For the PD-L1 positive ESCC patients, ORR (22.0 vs 7.0%), DOR (9.3 vs. 7.7 months), mPFS (3.2 vs. 2.3 months; HR = 0.66, 95% CI: 0.48–0.92), and mOS (10.3 vs. 6.7, HR = 0.62, 95% CI: 0.46–0.90) were better in the pembrolizumab group compared with chemotherapy group. Importantly, the incidence of trAEs in the pembrolizumab group was lower than the chemotherapy group (Any grade, 64.3 vs. 86.1%; grade 3–5, 18.2 vs. 40.9%). And there were five treatment-related deaths in each group.

##### Camrelizumab

Camrelizumab is a novel humanized high-affinity IgG4-kappa PD-1 monoclonal antibody that independently developed by Chinese biopharma. In the phase I study, NCT02742935, 30 ESCC patients failed to at least one systemic treatment were enrolled (48). Twenty-one (70.0%) patients had received two or more previous chemotherapy, 19 (63.3%) patients had radiation and 14 (46.7%) patients had esophagectomy. ORR was 33.3% (11/30), and mPFS was 3.6 months (95% CI: 0–7.2 months). The trAEs occurred in 25 (83.3%) patients and reactive capillary hemangiomas was the most frequent trAE (76.7%, 23/30), which was likely caused by activating the vascular endothelial growth factor (VEGF)/VEGF receptor pathway. Three (10.0%) grade 3 trAEs were reported: two (6.7%) pneumonitis and one (3.3%) increased cardiac troponin I. No grade 4–5 trAEs and no discontinuation because of trAEs in this trial.

The phase III trial, ESCORT study (NCT03099382), compared camrelizumab and chemotherapy (docetaxel or irinotecan) as the second-line treatment for advanced ESCC. The latest results were reported at the 15th OESO World Conference as an oral presentation (49). In the 448 enrolled patients, 228 patients were randomized to the camrelizumab group and reached an ORR of 20.2% and a 12-month survival rate of 33.7%. The mOS of camrelizumab group was better than the chemotherapy group (8.3 vs. 6.2 months; HR = 0.71, 95% CI: 0.57–0.87, *p* = 0.001).

##### Toripalimab

Toripalimab is another humanized PD-1 monoclonal antibody developed by Chinese biopharma. The latest data of a phase Ib/II trial, NCT02915432, was presented on the 2019 ASCO meeting (50). In this study, 59 advanced chemo-refractory ESCC patients were treated with toripalimab and the ORR was 18.6% (one CR and ten PR), mDOR was 11.2 months. Grade 3–5 trAEs occurred in 30.5% (18/59) of patients.

Based on these studies, pembrolizumab has been approved by the Food and Drug Administration (FDA) as the second-line treatment for recurrent, locally advanced or metastatic ESCC with PD-L1 positive (CPS ≥ 10) (51). It has also been approved as the third-line or subsequent therapy option for esophageal and EGJ adenocarcinomas with PD-L1 positive (CPS ≥ 1) before. Generous clinical trials are investigating the role of PD-1/PD-L1 inhibitors in advanced EC as subsequent treatment (durvalumab: NCT01938612, NCT02639065; pembrolizumab: NCT02971956, NCT02998268; CS1001: NCT03312842, NCT03744403) or as second-line treatment (sintilimab: NCT03116152; tislelizumab: NCT03430843, toripalimab: NCT03474640; pembrolizumab: NCT03933449). With previous studies showing its efficacy in ESCC patients, nivolumab has yet to secure FDA approval.

#### Combination With Immunoregulatory Factors

To boost the efficacy of PD-1/PD-L1 inhibitors in EC patients, we concentrate on its combination with different immunoregulatory factors to activate anti-tumor immunity (Table 5).

**Table 5:** The PD-1/PD-L1 inhibitors combined with immunoregulatory factors.

**Target**	**Drug**	**Trials**
CTLA-4 inhibitor	Ipilimumab	NCT03416244
LAG-3 inhibitor	Relatlimab, LAG525	NCT03044613, NCT02460224
OX40 agonist	INCAGN01949	NCT03241173
GITR agonist	INCAGN01876	NCT03277352
ICOS inhibitor	KY1044	NCT03829501
IDO1 inhibitor	Epacadostat	NCT03592407, NCT03277352
CCR-4 inhibitor	Mogamulizumab	NCT02476123, NCT02946671
M-CSF inhibitor	MCS110	NCT03785496
WNT inhibitor	LGK974	NCT01351103
DKK-1 inhibitor	DKN-01	NCT02013154, NCT03818997
PDE-5 inhibitor	Tadalafil	NCT03993353
SHP2 inhibitor	TNO155	NCT04000529

##### LAG-3

Lymphocyte activation gene-3 (LAG-3, CD223) is regularly expressed on activated T cells and natural killer (NK) cells. The expression and upregulation of LAG-3 and PD-1 on tumor-infiltrating lymphocytes (TILs) leading to the inactivate of effector T cells and cause tumor growth (52). Relatlimab (BMS-986016) and LAG525 are humanized monoclonal anti-LAG-3 antibodies. LAG-3 inhibitors hinder the interaction of LAG-3 with MHC class II and then repair the activity of effector T cells to kill tumor cells. The combination of PD-1/PD-L1 inhibitors and anti-LAG-3 antibody is under investigation now (NCT03044613, NCT02460224) and is expected to produce a synergistic effect.

##### OX40

OX40 (CD134) is a T cell co-stimulatory receptor and mainly expressed on activated T cells, and regulatory T (Treg) cells (53). The binding of OX40 to OX40L provokes the activation and proliferation of T cells, enhances effector T-cell differentiation and decreases the immunosuppressive function of Treg cells. INCAGN01949 is a humanized immunoglobulin G1 agonistic monoclonal antibody of OX40 and is being judged in EC patients (NCT03241173).

##### IDO1

Indoleamine 2.3-dioxygenase-1 (IDO1) is an intracellular enzyme and overexpressed in various cancers. As a metabolic mediator, IDO1 enzyme transfer tryptophan to tryptophan catabolites (54). This conversion lessens the activity of effector T cells and promotes the differentiation and function of Treg through upregulating FoxP3. Epacadostat is an oral IDO inhibitor and is being investigated with PD-1/PD-L1 inhibitors (NCT03592407, NCT03277352).

There are also novel multi-target antibodies, including SL-279252 (PD-1 and OX40L inhibitor) and INBRX-105 [PD-L1 and 41BB (CD137) inhibitor]. The safety and efficacy of these agents may be reported in forthcoming studies.

## Prospect: Identify Beneficial Patients

Considering the low effective rate of ICI in EC, predictive biomarkers are needed to decide patients more likely to react to ICI and identify patients resistant to ICI and need a combination or alter treatment. Predictive biomarkers identified in NSCLC including PD-L1 status, tumor mutation burden (TMB), mismatch repair deficiency (MMR), and microsatellite instability (MSI). Whether they make a similar role in EC is uncertain.

### Pathological Types and Ethnic Difference

As mentioned before, ESCC is more widespread in Asia, and clinical trials reveal higher ORRs in ESCC patients with PD-1/PD-L1 inhibitors compared with EAC. ATTRACTION-1 reported an ORR of 17% (95% CI: 10–28%) in 65 advanced Japanese ESCC patients treated with nivolumab (43). KEYNOTE-028 enrolled 23 EC patients and found that ORR was 28% in ESCC and 40% in EAC patients with pembrolizumab treatment, respectively (12). However, the large sample trial KEYNOTE-180 showed that the ORR for patients with pembrolizumab was 14.3% (95% CI: 6.7–25.4%) in ESCC and 5.2% (95% CI: 1.1–14.4%) in EAC patients (46). Furthermore, the KEYNOTE-181 trial revealed that in all PD-L1 positive patients, ESCC ones might have better ORR (22.0 vs. 18.0%), mPFS (3.2 vs. 2.1 months), and mOS (10.3 vs. 6.3 months) than EAC ones (47). There was no direct comparison between ESCC and EAC patients in these studies.

Regarding the better response to PD-1/PD-L1 in ESCC, Asian patients may be the more beneficial population. The latest results of Chinese patients in KEYNOTE-181 were presented on the 2019 ESMO meeting (55). Among the 123 enrolled advanced EC patients, 119 had ESCC and 54 had PD-L1 positive. In the 62 patients treated with pembrolizumab, ORR was 16.1% and mDOR was not reached (4.4+ to 14.6+ months). The mOS was longer in the pembrolizumab group in all the Chinese patients (8.4 vs. 5.6 months; HR = 0.55, 95% CI: 0.36–0.82), in the ESCC (8.4 vs. 5.6 months; HR = 0.55, 95% CI: 0.37–0.83), and in the PD-L1 positive patients (12.0 vs. 5.3 months; HR = 0.34, 95% CI: 0.17–0.69). Chinese patients perhaps have better mOS, but the comparison with other patients was lacked.

Thus, Asian and non-Asian patients may have different efficacy even within ESCC. Further analysis is needed to help draw an accurate conclusion.

### PD-L1 Status and TILs

The predictive role of PD-L1 is still controversial in EC. Some studies believe that PD-L1 expression in tumor and immune cells is associated with the efficacy of PD-1/PD-L1 inhibitors (56). In the KEYNOTE-180 trial, patients with PD-L1 positive (CPS ≥ 10) had better ORR (13.8 vs. 6.35%) when treating with pembrolizumab (57). This was also supported by the trial KEYNOTE-181, which showed that pembrolizumab could significantly improve OS compared with chemotherapy in PD-L1 positive (CPS ≥ 10) EC patients (mOS: 9.3 vs. 6.7 months; HR = 0.69, 95% CI: 0.52–0.93, *p* = 0.0074) (47). However, PD-L1 status was not significantly related to ORR and DCR in the trial NCT02742935 of camrelizumab in Chinese ESCC (48). Besides, in another clinical trial of toripalimab (NCT02915432), the PD-L1 status was also not a predictive biomarker for clinical benefit in Chinese ESCC patients (58).

In some PD-L1 positive patients, the efficacy of PD-1/PD-L1 inhibitors is still low. Besides the heterogeneity of PD-L1 expression, TILs may be the possible reason. TILs consist of a group of heterogenous lymphocytes that infiltrate the tumor and participated in anti-tumor response. The high level of TILs in TME is correlated to better survival in patients with EC (59), NSCLC (60), breast cancer (61), and so on. Furthermore, TILs were also associated with the clinical benefit from PD-1/PD-L1 inhibitors in melanoma (62). In KEYNOTE-001 (NCT01295827) study, patients of melanoma were treated with pembrolizumab. CD8+ T-cell densities were higher in the pretreatment tumor samples of responding patients (63). In another study of patients with melanoma, CD8+, CD3+, and CD45RO+ T-cell densities in pretreatment samples were associated with response to PD-1 inhibitor (64). However, the predictive value of TILs and the most important cells in TILs are still unknown for EC so far.

The relationship of PD-L1 status and the efficacy of PD-1/PD-L1 inhibitors remains uncertain, and the function of other factors in TME still needed to be considered and provide more evidence in the future.

### TMB, MSI, and dMMR

TMB is the number of non-synonymous somatic gene mutations (Mb) of sequenced DNA and higher TMB tumors are likely to produce more neoantigens, induce a specific T cell response, and further enhance the anti-tumor immunity. High TMB correlates with clinical benefit from ICIs in patients with melanoma and NSCLC (65, 66).

The number of TMB varies in different cancers. TMB is low in EC according to the reports of patients from western countries. (67). However, the analysis in Chinese EC patients showed higher TMB (68). The expression of TMB in EC is unclear, and the proper cutoff value of high TMB is also undecided. In phase Ib/II trial, NCT02915432, chemo-refractory ESSC patients received toripalimab and 11 (23.4%) patients with high TMB (≥12 Mutations/Mb) showed no significant advantage in ORR or OS (50). More studies are required to judge the role of TMB in EC patients and the proper cutoff value of high TMB.

Mismatch repair genes are genes that replace nucleotides incorrectly incorporated during DNA replication. Deficient DNA mismatch repair (dMMR) means a lack of these genes and produce a lot of short repeated sequences in the DNA (microsatellite) and more tumor-specific mutation (higher TMB) (69). MSI can be divided into high (MSI-H), low (MSI-L) and stable (MS-S). So far, NCCN guidelines have recommended pembrolizumab as the second-line or subsequent treatment for MSI-H or dMMR solid cancers, including EC (25). Although dMMR or MSI-H are predictive biomarkers for PD-1/PD-L1 inhibitors, this appears to be more of a gastric cancer phenomenon and only occurs in about 8% of EC patients (69).

### Other Predictive Biomarkers

Despite the potential biomarkers mentioned above, many different predictive biomarkers are also studied now. The trial NCT02915432 analyzed the amplification of the chromosome 11q13 region in ESSC patients received toripalimab. Forty-eight percentage (24/50) patients had 11q13 amplification, which resulted in elevated mRNA expression of corresponding genes, including Cyclin D1 (CCND1) and fibroblast growth factor family members (FGF3/4/19) (50). Patients without 11q13 amplification, had considerably better ORR (30.8 vs. 4.2%, *p* = 0.024) and mPFS (3.7 vs. 2.0 months; HR = 0.47, 95% CI: 0.24–0.91, *p* = 0.025).

In another trial about camrelizumab, ESCC patients with an increased baseline lactate dehydrogenase (LDH) had lower ORR (*p* = 0.02) and shorter PFS (*p* = 0.002) and OS (*p* < 0.0001) than patients with normal LDH (NCT02742935) (70). Meanwhile, the increase of LDH during treatment was related to disease progression. Multivariate Cox analysis shown that LDH (HR = 0.18), C-reactive protein (CRP) (HR = 0.27), the number of organs involved (HR = 0.31), absolute monocyte count (HR =0.33), and Eastern Cooperative Oncology Group (ECOG) performance status (HR = 0.36) are independent prognostic factors in this trial.

Currently, the predictive role of a single biomarker is limited, combined prediction models of multiple biomarkers may be available in the future.

## Conclusion

Based on the previous results, PD-1/PD-L1 inhibitors were durable and effective in EC, though many questions remain unanswered. Firstly, most trials are single-arm designed. More randomized controlled trials are demanded to compare the efficacy of PD-1/PD-L1 inhibitors and control treatment. Secondly, the control treatment in the current studies was chemotherapy alone rather than other more effective therapies, such as CRT. Thirdly, the response rates of PD-1/PD-L1 inhibitors alone were limited. Its combination with chemotherapy, RT, targeted drugs or other immune modulates may improve the anti-tumor activity. It is extremely important to identify patients who most likely gain clinical benefit from PD-1/PD-L1 inhibitors. More predictive biomarkers are investigated to refine the optimal patient for single-agent treatment and those require combination therapies. We also include the AEs of PD-1/PD-L1 inhibitor alone or combined with others, especially the incidence of pneumonitis.

## Author Contributions

HY and LY designed the study. HY collected data of clinical trials and drafted the manuscript together with KW. TW, ML, BL, and SL coordinated, edited, and completed the drafting of the manuscript. LY revised and edited the final version of the manuscript for important intellectual content. All authors read and approved the final manuscript.

### Conflict of Interest

The authors declare that the research was conducted in the absence of any commercial or financial relationships that could be construed as a potential conflict of interest.

## Footnotes

^1^https://www.agilent.com/en/product/pharmdx/pd-l1-ihc-22c3-pharmdx/pd-l1-ihc-22c3-pharmdx-for-autostainer-link-48-94448

^2^https://www.agilent.com/en/product/pharmdx/pd-l1-ihc-28-8-pharmdx/pd-l1-ihc-28-8-pharmdx-for-autostainer-link-48-76917

^3^https://diagnostics.roche.com/global/en/products/tests/ventana-pd-l1-_sp142-assay1.html

^4^https://diagnostics.roche.com/global/en/products/tests/ventana-pd-l1-_sp263-assay1.html
