# Supplementary material for: The Combination Options and Predictive Biomarkers of PD-1/PD-L1 Inhibitors in Esophageal Cancer
Source: Front Oncol. 2020 Mar 5;10:300. doi: 10.3389/fonc.2020.00300 (PMC7066251; doi:10.3389/fonc.2020.00300)
Supplement: Supplementary file 1 [file Table_1.pdf]

Table S1. The detailed information of published trials

Study	Phase	Design	Publication form	Reference
As neoadjuvant treatment				
NCT03044613	II/III	Non-randomized, single-arm, open-label	Conference abstract	22
NCT02844075	II	Single-arm, open-label	Conference abstract	23
NCT03490292	I/II	Non-randomized, single-arm, open-label	Conference abstract	24
NCT03087864	II/III	Single-arm, open-label	Conference abstract	25
As adjuvant treatment				
NCT02639065	II	Single-arm, open-label, multi-center	Conference abstract	35
As first-line treatment				
NCT03187314	II	Single-arm, open-label	Conference abstract	37
NCT03222440	Ib	Non-randomized, single-arm, open-label, single center	Conference abstract	38
NCT03469557	II	Non-randomized, single-arm, open-label	Conference abstract	41
NCT02658214	Ib	Non-randomized, single-arm, open-label	Conference abstract	43
NCT03603756	II	Non-randomized, single-arm, open-label	Conference abstract	44
As second-line or subsequent treatment				
ATTRACTION-1	II	Single-arm, open-label, multi-center	Full text	47
ATTRACTION-3 (NCT02569242)	III	Randomized, controlled, open-label, multi-center	Full text and conference abstract	48, 49
KEYNOTE-028 (NCT02054806)	Ib	Single-arm, open-label	Full text	16
KEYNOTE-180 (NCT02559687)	II	Single-arm, open-label	Conference abstract	50
KEYNOTE-181 (NCT02564263)	III	Randomized, controlled, open-label	Conference abstract	51, 52
NCT02742935	I	Non-randomized, single-arm, open-label, single center	Conference abstract	53
NCT03099382	III	Randomized, controlled, open-label	Conference oral presentation	54
NCT02915432	Ib/II	Non-randomized, single-arm, open-label, multi-center	Conference abstract	55
